# Microstructure Images Restoration of Metallic Materials Based upon KSVD and Smoothing Penalty Sparse Representation Approach

**DOI:** 10.3390/ma11040637

**Published:** 2018-04-20

**Authors:** Qing Li, Steven Y. Liang

**Affiliations:** 1 College of Mechanical Engineering, Donghua University, Shanghai 201620, China; steven.liang@me.gatech.edu; 2 George W. Woodruff School of Mechanical Engineering, Georgia Institute of Technology, Atlanta, GA 30332-0405, USA

**Keywords:** image restoration, KSVD dictionary, smoothing penalty sparse representation (SPSR), microstructure images, aluminum alloy 7075 (AA7075) material

## Abstract

Microstructure images of metallic materials play a significant role in industrial applications. To address image degradation problem of metallic materials, a novel image restoration technique based on K-means singular value decomposition (KSVD) and smoothing penalty sparse representation (SPSR) algorithm is proposed in this work, the microstructure images of aluminum alloy 7075 (AA7075) material are used as examples. To begin with, to reflect the detail structure characteristics of the damaged image, the KSVD dictionary is introduced to substitute the traditional sparse transform basis (TSTB) for sparse representation. Then, due to the image restoration, modeling belongs to a highly underdetermined equation, and traditional sparse reconstruction methods may cause instability and obvious artifacts in the reconstructed images, especially reconstructed image with many smooth regions and the noise level is strong, thus the SPSR (here, *q* = 0.5) algorithm is designed to reconstruct the damaged image. The results of simulation and two practical cases demonstrate that the proposed method has superior performance compared with some state-of-the-art methods in terms of restoration performance factors and visual quality. Meanwhile, the grain size parameters and grain boundaries of microstructure image are discussed before and after they are restored by proposed method.

## 1. Introduction

Microstructure images of metallic materials play a significant role in industrial applications, such as metallography detection, surface topography measurement (STM), and micro-electro mechanical manufacturing (MEMM) [1,2,3], etc. However, the raw microstructure images are easily contaminated on their acquisition, storage, and transmission, which degrade the fidelity of the microstructure image. Further, in most cases, images re-cquisition is not possible in practice, as such damaged images are not suitable for subsequent processing [4,5].

Image restoration is an effective technique for recovering incomplete or damaged images approximate to the ideal images. Up to now, various image restoration strategies have been proposed, which can be classified into several categories: (i) Time domain analysis methods, such as adaptive filter denoising (AFD) [6,7]; (ii) Frequency domain analysis methods, such as wavelet–wavelet packet denoising (W-WPD) technique [8,9]; (iii) Data-driven approaches, such as partial differential equation (PDE) [10,11,12,13], and wavelet hidden Markov random field (WHMRF) [14]; (iv) Sparse representation (SR) techniques, such as redundant dictionary and non-convex penalty regularization [15,16,17,18], etc. Although the damaged images can be restored more precisely by the above methods, the drawbacks are also obvious. For example, the adaptive filter denoising method can achieve a good denoising effect under low noise level, but the denoising effect will be greatly reduced with the noise increases. The W-WPD can improve the quality of image, but some artifacts (e.g., ambiguity points) might be generated along with the reconstruction process. The main shortcomings, including optimal parameter selection and high computation cost, still remain unsolved in data-driven approaches. For the WHMRF method, it is difficult to flexibly integrate the spatial quantitative relation (between the damaged pixel point and its neighbor points) into the restoration model [19].

Today, SR techniques are based on the principle that an image can be sparsely represented in a time domain or frequency transform domain, where each image block could be reconstructed by sparse reconstructing algorithms. The core idea behind SR is how to represent the images more sparsely in the time/frequency transform domains, usually, the most common methods focus on the establishment of redundant multiscale transform and redundant dictionary. For example, in ref. [20], the curvelet transform is proposed to denoise the white noise, which exhibits higher perceptual quality than wavelet-based reconstructions. In ref. [21], the edge detection and fuzzy clustering algorithm are combined to preserve the edges of synthetic aperture radar (SAR) despeckling in translation-invariant second-generation band-wavelet transform (TIBT) domain. In ref. [22], to address the image denoising problem, the over-complete discrete cosine transform (DCT) dictionary, global trained dictionary, and K-means singular value decomposition (KSVD) dictionary, were proposed.

Another core point of the SR is reconstruction algorithm. Generally, image restoration is an inverse problem, in which the ideal images could be approximatively estimated from the noisy images. The difficulty knot of image restoration is that the inverse problems are often ill-posed (or non-deterministic polynomial-time hard, NP-hard). In traditional sparse representations, most of the methods are applied based on regularization-based technique, for example, matching pursuit (MP) [23], orthogonal matching pursuit (OMP) [24], and compressive sampling matching pursuit (CoSaMP) [25], some regularization models, such as total variation regularization function (TVRF) [26], sparse non-local regularization (SNLR) [27,28], etc. However, the traditional sparse representation methods may cause instability and obvious artifacts in the reconstructed images, especially for the restoration of microstructure image that include some smooth regions and multi-boundary and fine-textures, or when the noise level is strong.

To address the above issues in SR and exploit the spatial information of microstructure images, in this paper, a novel image restoration method based upon smoothing penalty sparse representation (SPSR) and adaptive over-complete KSVD dictionary is proposed for microstructure image of metallic materials, taking aluminum alloy 7075 (AA7075) material as an example. The nonconvex penalty regularization is introduced to address the image inverse problem mentioned above, and the success rate will be improved greatly in image reconstruction. Moreover, unlike the common procedure used in refs. [29,30], in which the sparse transform basis was used for measuring the image sparsity, this paper utilizes over-complete dictionary (e.g., trained KSVD dictionary) to promote the image sparsity under the given redundant system. The simulation and experimental results show the superiority of the proposed method compared with some state-of-the-art methods, such as wavelet packet method, the discrete cosine transformation (DCT) combined with OMP method, and the KSVD dictionary combined with OMP method, respectively. Meanwhile, the comparison results of grain parameters, such as grain diameter (mean), grain area (polygon), grain perimeter (ratio), etc., are also discussed in detail before and after processing based on the proposed method.

For the applicability of the proposed method, generally, a microstructure image is used that exhibits a lamellar (plate-like) structure or a structure that exhibits twinning, which can be divided into two kinds of regions: one is the image blocks containing lamellar (plate-like) structure and boundaries in structure regions, etc., and the other is the image blocks that distributed in smooth regions. Correspondingly, the optimization expression of the sparse representation method usually includes a likelihood part and prior knowledge part, furthermore, the external noise will play a critical role in smooth regions during image restoration, and its effect is relatively weak in structured regions, where there is a strong similarity between the two blocks, due to the pixel values being similar in smooth regions, and if the noise level is strong, the information characteristics of external noise in smooth regions might be regarded as structural information in sparse coefficients, wherein the classical optimization approach might remove the inveracious structure information that failed, leading to instability and obvious artifacts on the reconstructed image. For the proposed method, on the one hand, before the reconstruction algorithm is implemented, the information of lamellar (plate-like) structure and boundaries could be represented in over-complete KSVD trained dictionary, on the other hand, the prior knowledge part is improved by introducing a smoothing parameter, and the likelihood part is also improved via a regularization weight, therefore, the noise distributed in structure regions and smooth regions could be denoised adaptively, especially for the noise points hidden in the smooth regions.

The main contributions of this paper are summarized as follows:

(1) The dictionary training algorithm, namely KSVD, is introduced, while the detailed structure information, such as lamellar (plate-like) structure and boundaries, could be represented accurately.
(2) The smoothing parameter and regularization weight are designed based upon the traditional sparse representation method, and the noise distributed in structure regions and smooth regions could be denoised adaptively.
(3) The grain parameters, such as grain diameter (mean), grain area (polygon), grain perimeter (ratio), etc., are discussed before and after they are processed by the proposed method, and the structural information that used for industrial applications, such as metallography detection, micro-electro mechanical manufacturing (MEMM) could be clearly isolated, which may open up a new field of application of material microstructure to industry.

The layout of this paper is organized as follows. Section 2 describes the algorithms of sparse representation and KSVD. Section 3 introduces smoothing penalty sparse representation (SPSR) algorithm, and its derivation and parameter selection, etc. In Section 4, the simulation and experimental results of the proposed method are presented with other approaches. Conclusions are shown in Section 5.

## 2. Sparse Representation and KSVD Algorithm

### 2.1. The Review of Sparse Representation (SR)

Generally, the image degradation process can be described by a degradation matrix ***R*** as follows:

(1) y=Rx+n

where *y* is the damaged image, *x* is the latent ideal image and *n* is external noise. Usually, image *x* is assumed to be *K*-sparse, i.e., which having *K* non-zero components (the pixel value of the measurement matrix in the damaged area is 0, in contrast, undamaged area is 1).

According to the framework of compressed sensing (CS) [31,32], a discrete 1-D signal with limited length *x*_0_ that can be represented in the domain $\varphi =[\varphi_{1},\varphi_{2},\ldots ,\varphi_{N}]$,

(2) $$x_{0}=\sum_{i=1}^{N}\varphi_{i}\alpha_{i}=\varphi \alpha$$

where α is the transformation coefficients of signal *x* in domain φ. Usually, the commonly used sparse transformation base φ includes discrete wavelet transform (DWT) basis, discrete cosine transform (DCT) basis, Gabor basis and curvelet basis, etc. [33]. Further, if the 1-D discrete signal is expanded into 2-D image, i.e., $x_{0}\to x$, Equation (1) can now be rewritten as follows:

(3) y=Rx+n=Rφα+n=Aα+n

where A=Rφ denotes sensing matrix. Here, Candes et al. [32,34,35] showed that the Equation (3) can only be solved when the sensing matrix ***A*** satisfies the conditions of incoherence or restricted isometry property (RIP), where the RIP is given by

(4) $$(1-\delta ){\left\|\alpha \right\|}_{2}\leq {\left\|A\alpha \right\|}_{2}\leq (1+\delta ){\left\|\alpha \right\|}_{2}$$

where parameter δ controls the level of data discrepancy based on an estimation of the noise variance. Usually, the common measurement matrix includes Gaussian random matrix (GRM), local Fourier matrix (LFM) and local Hadamard matrix (LHM), etc.

### 2.2. KSVD Algorithm

To enhance the sparsity of the damaged image, in this section, the adaptive over-complete dictionary is employed to substitute the traditional sparse transform basis, that is, the image blocks will be represented by trained KSVD method [36], i.e., A=RD. It should be noted that the matrix ***A*** may not satisfy the RIP criterion, due to the over-complete dictionary ***D***; to address this issue, the mutual coherence technique [37] is applied to substitute the matrix ***A***, i.e.,

(5) $$\mu (A)=\underset{i\neq j}{\max}\frac{\left|<\alpha_{i},\alpha_{j}>\right|}{{\left\|\alpha_{i}\right\|}_{2}{\left\|\alpha_{j}\right\|}_{2}},i,j=1,2,\ldots ,K$$

where $\alpha_{i}$ and $\alpha_{j}$ are the *i*-th and *j*-th column in matrix ***A***, respectively. The adaptive over-complete KSVD dictionary method is designed as follows:

Step (1). Dictionary initialization. Suppose that $D\in {\mathbb{R}}^{n\times k}(k<<n)$ is the over-complete dictionary, for example, over-complete discrete cosine transform (DCT) dictionary.

Step (2). Dictionary presentation. Suppose that $D\in {\mathbb{R}}^{n\times k}(k<<n)$ is the over-complete DCT dictionary, $Y={\left\{y_{i}\right\}}_{i=1}^{N}$ is a *N*-training samples set and $X={\left\{x_{i}\right\}}_{i=1}^{N}$ is a solution vectors set of ***Y***, hence,

(6) $$\underset{D,X}{\min}{\left\|Y-DX\right\|}_{F}^{2},\;s.\;t.,\;\forall i,{\left\|x_{i}\right\|}_{0}\leq T_{0},\;i=1,2,\ldots ,N$$

where *T*_0_ is the maximum value of nonzero vector in sparse coefficients and ${\left\|\cdot \right\|}_{F}$ is Frobenius norm.

Step (3). Dictionary updated. Assume that $d_{k}$ refer to the *k*-th column of the pending update dictionary, then Equation (6) equivalent to

(7) $${\left\|Y-DX\right\|}_{F}^{2}={\left\|(Y-\sum_{j\neq k}^{K}d_{k}x_{T}^{j})-d_{k}x_{T}^{k}\right\|}_{F}^{2}={\left\|E_{k}-d_{k}x_{T}^{k}\right\|}_{F}^{2}$$

where ***E**_k_* represents pre-computed error matrix, $x_{T}^{k}$ represents the *k*-th row of *X* which actually gives the sparse coefficients or weights of $d_{k}$. For the purpose of singular value decomposition (SVD), four parameters are defined as follows:

(8) $$\left\{ \begin{array}{l} \omega_{k}=\{i|1\leq i\leq K,x_{T}^{k}(i)\neq 0\},\\ x_{R}^{k}=x_{T}^{k}\Omega_{k},\\ Y_{k}^{R}=Y\Omega_{k},\\ E_{k}^{R}=E_{k}\Omega_{k}, \end{array}\right.$$

where $\Omega_{k}$ is a matrix with size $N\times \left|\omega_{k}\right|$. The parameter $Y_{k}^{R}$ includes a subset of the samples that are currently using $d_{k}$ element, $E_{k}^{R}$ represents error columns that correspond to samples that using $d_{k}$ element, respectively. Returning to the objective Equation (7), it can be rewritten as

(9) $${\left\|E_{k}\Omega_{k}-d_{k}x_{T}^{k}\Omega_{k}\right\|}_{F}^{2}={\left\|E_{k}^{R}-d_{k}x_{R}^{k}\right\|}_{F}^{2}$$

Step (4). Singular value decomposition. The ***E**_k_* is decomposed by SVD method, i.e., $E_{k}=U\Delta V^{T}$, $\overset{\wedge }{d_{k}}$ is the first column updated by $d_{k}$ in ***U***, then the second column will be updated successively until the last column, otherwise, go back to step (3), and the new trained dictionary $\overset{\wedge }{D}$ will be finally obtained.

## 3. Smoothing Penalty Sparse Representation (SPSR)

It should be noted that y=Aα+n belongs to a highly underdetermined equation (HUE) [38,39], there are infinite set of solutions. The problem of image reconstruction by sparse representation under residual error constraint can be calculated by

(10) $$\tilde{\alpha }=\arg\min{\left\|\alpha \right\|}_{0},\;s.\;t.,\;{\left\|A\alpha -y\right\|}_{2}^{2}\leq c$$

where *c* is a threshold of the residual error. Moreover, the prior knowledge of the original image is usually utilized to regularize the solution under residual error constraint is expressed as

(11) $$\tilde{\alpha }=\arg\min{\left\|A\alpha -y\right\|}_{2}^{2}+\lambda \cdot \zeta (x)$$

where λ is regularization weight and ζ(x) regularization term. From the perspective of Bayesian estimation, the ${\left\|A\alpha -y\right\|}_{2}^{2}$ and ζ(x) can be viewed as the likelihood part and prior knowledge part, respectively. Therefore, the ζ(x) prior knowledge plays a significant role in image restoration based on sparse representation. For a microstructure image, it can also be divided into two types: the first is the image blocks containing details, boundaries, and singular points, and the second is the image blocks located in smooth regions. For the former, the details, boundaries, and singular points that determine the similarity between two blocks, and the influence of external noise, is relatively weak. However, in smooth regions, there is a strong similarity between the two blocks due to the pixel values being similar; here the influence of external noise will play a critical role in image restoration. If the noise level is strong, the information of noise in smooth regions is regarded as structural information in sparse coefficients. Meanwhile, the classical optimization approaches and regularization approaches cannot remove the false structural information contained in the sparse coefficients, and the traditional methods may cause instability and obvious artifacts in the reconstructed images.

To address this issue, a novel smoothing penalty sparse representation (SPSR) method is introduced, that is,

(12) $$L_{q}(\alpha ,\varepsilon ,\lambda )=\sum_{j=1}^{N}{\left[\alpha_{j}^{2}+\varepsilon^{2}\right]}^{q/2}+\frac{1}{2\lambda }{\left\|A\alpha -b\right\|}_{2}^{2}$$

where *q* is regular operator, ε(ε>0) is smoothing parameter, λ(λ>0) is penalty parameter, and *b* is measurement vector. It should be mentioned that the smoothing parameter ε(ε>0) plays a critical role in image restoration in terms of smooth regions. The detailed updated procedures of the proposed Algorithm 1 are as follows:
**Algorithm 1.** The smoothing penalty sparse representation algorithm.**SPSR (0 < *q* ≤ 1) algorithm:****Input**: Matrix ***A***, measurement vector *b*, estimated sparsity level *s*.**Output**: Recovery vector α;(1) Choose appropriate parameters λ(λ>0), *q* (0 < *q* ≤ 1);(2) Initialize $\alpha^{\left(0\right)}$, such that $A\alpha^{\left(0\right)}=b$, and set $\omega^{\left(0\right)}=e^{0}$ and $\varepsilon_{0}=1$;(3) For *k* = 0;(4) Solve the following linear system for $\alpha^{\left(k\right)}$,${\left[\frac{q\alpha^{\left(k+1\right)}[i]}{{\left(\varepsilon_{k}^{2}+{\left\|\alpha^{\left(k\right)}[i]\right\|}_{2}^{2}\right)}^{1-\frac{q}{2}}}\right]}_{1\leq i\leq M}+\frac{1}{\lambda }A^{T}(A\alpha^{\left(k+1\right)}-b)=0$Or$(A^{T}A+diag{\left(\frac{q\lambda }{{\left(\varepsilon_{k}^{2}+{\left\|\alpha^{\left(k\right)}[i]\right\|}_{2}^{2}\right)}^{1-\frac{q}{2}}}\right)}_{1\leq i\leq M})\alpha^{\left(k+1\right)}=A^{T}b$(5) When $\alpha^{\left(k\right)}$ meet the reconstruction accuracy, $\alpha^{\left(k\right)}$ as the output value assigned to α, meanwhile end to this algorithm, otherwise execute next steps.(6) Let β is a constant, where (0<β<1), update $\varepsilon_{k+1}=\min\{\varepsilon_{k},\beta \cdot r{\left(\alpha^{\left(k+1\right)}\right)}_{s+1}/M\}$, where r(α) represents the rearrangement of absolute values of $r(\alpha^{\left(k+1\right)})$ in the decreasing order, and $r{\left(\alpha \right)}_{s+1}$ is the (*s* + 1)th component value of r(α). Note that, if $\varepsilon_{k+1}=0$, choose $\alpha^{\left(k+1\right)}$ to be an approximation of sparse solution and stop this iteration.(7) Let *k* = *k* + 1, and return to Step (4) to continue.**End**

For the SPSR method, the following theorem summarizes the results for 0 < *q* ≤ 1, thus, we have the following theorem which can prove the above proposed algorithm.

**Theorem** **1.** *Error estimation theorem [40]. Suppose that x^0^ is s-sparse signal which satisfies **A**x^0^ = b. The smooth parameter $\varepsilon_{k}\to \varepsilon_{*}$ with k→∞. Matrix **A** satisfies the RIP of order 2s with $\delta_{2s}<1$, when $\varepsilon_{*}>0$, the sequence {x^(k)^} has at least one convergent subsequence. Suppose that the limit $\varepsilon_{k}=\varepsilon_{*}$ is a local optimal solution for Equation (12), we have,*

(13) $${\left\|x^{\varepsilon_{*}}-x^{0}\right\|}_{2}\leq C_{1}\sqrt{\lambda }+C_{2}\delta_{s}{\left(x^{\varepsilon_{*}}\right)}_{2}$$

*where $\delta_{s}{\left(x^{\varepsilon_{*}}\right)}_{2}$ is the approximate error of $x^{\varepsilon_{*}}$, which satisfies $\delta_{s}{\left(x^{\varepsilon_{*}}\right)}_{2}=\underset{{\left\|y\right\|}_{2,0}\leq s}{\inf}{\left\|x^{\varepsilon_{*}}-y\right\|}_{2}$. For the special case, when $\varepsilon_{*}=0$, there must exist a convergent subsequence converging to point x^0^, it satisfies,*

(14) $${\left\|x^{0}-x^{*}\right\|}_{2}\leq C_{3}\sqrt{\lambda }$$

*where C_1_, C_2_ are C_3_ are independent positive constants. To prove the Theorem 1, the following two lemmas are required*.

**Lemma** **1.** *For all $x,y\in R^{N}$ and 0 < q ≤ 1, if $\varepsilon_{k}\geq \varepsilon_{k+1}\geq 0$, it satisfies,*

(15) $${\left(\varepsilon_{k}^{2}+{\left\|x\right\|}_{2}^{2}\right)}^{\frac{q}{2}}-{\left(\varepsilon_{k+1}^{2}+{\left\|y\right\|}_{2}^{2}\right)}^{\frac{q}{2}}-\frac{qy^{T}(x-y)}{{\left(\varepsilon_{k}^{2}+{\left\|x\right\|}_{2}^{2}\right)}^{1-\frac{q}{2}}}\geq \frac{q{\left\|x-y\right\|}_{2}^{2}}{2{\left(\varepsilon_{k}^{2}+{\left\|x\right\|}_{2}^{2}\right)}^{1-\frac{q}{2}}}$$

**Proof.**  According to arithmetic-geometric mean inequality [41], i.e.,

(16) $${\left(\varepsilon_{k}^{2}+{\left\|x\right\|}_{2}^{2}\right)}^{1-\frac{q}{2}}{\left(\varepsilon_{k+1}^{2}+{\left\|y\right\|}_{2}^{2}\right)}^{\frac{q}{2}}\leq (1-\frac{q}{2})(\varepsilon_{k}+{\left\|x\right\|}_{2}^{2})+\frac{q}{2}(\varepsilon_{k+1}+{\left\|y\right\|}_{2}^{2})$$

Then we compute,

$$\begin{array}{l} {\left(\varepsilon_{k}^{2}+{\left\|x\right\|}_{2}^{2}\right)}^{\frac{q}{2}}-{\left(\varepsilon_{k+1}^{2}+{\left\|y\right\|}_{2}^{2}\right)}^{\frac{q}{2}}-\frac{qy^{T}(x-y)}{{\left(\varepsilon_{k}^{2}+{\left\|x\right\|}_{2}^{2}\right)}^{1-\frac{q}{2}}}\\ =\frac{\left(\varepsilon_{k}^{2}+{\left\|x\right\|}_{2}^{2})-{\left(\varepsilon_{k}^{2}+{\left\|x\right\|}_{2}^{2}\right)}^{1-\frac{q}{2}}{\left(\varepsilon_{k+1}^{2}+{\left\|y\right\|}_{2}^{2}\right)}^{\frac{q}{2}}-qy^{T}(x-y\right)}{{\left(\varepsilon_{k}^{2}+{\left\|x\right\|}_{2}^{2}\right)}^{1-\frac{q}{2}}}\\ \geq \frac{\left(\varepsilon_{k}^{2}+{\left\|x\right\|}_{2}^{2})-(1-\frac{q}{2})(\varepsilon_{k}^{2}+{\left\|x\right\|}_{2}^{2})-\frac{q}{2}(\varepsilon_{k+1}^{2}+{\left\|y\right\|}_{2}^{2})-qy^{T}(x-y\right)}{{\left(\varepsilon_{k}^{2}+{\left\|x\right\|}_{2}^{2}\right)}^{1-\frac{q}{2}}}\\ =\frac{q}{2}\frac{\varepsilon_{k}^{2}-\varepsilon_{k+1}^{2}+{\left(x-y\right)}^{2}}{{\left(\varepsilon_{k}^{2}+{\left\|x\right\|}_{2}^{2}\right)}^{1-\frac{q}{2}}}\geq \frac{q}{2}\frac{{\left(x-y\right)}^{2}}{{\left(\varepsilon_{k}^{2}+{\left\|x\right\|}_{2}^{2}\right)}^{1-\frac{q}{2}}} \end{array}$$

This completes the proof of Lemma 1. ☐

**Lemma** **2.** *Let $L_{q}(x,\varepsilon ,\lambda )=\sum_{j=1}^{N}{\left[\alpha_{j}^{2}+\varepsilon^{2}\right]}^{q/2}+\frac{1}{2\lambda }{\left\|A\alpha -b\right\|}_{2}^{2}$, if be the solution of $L_{q}(x,\varepsilon ,\lambda )$ for k = 0, 1, 2, … N, then,*

(17) $${\left\|Ax^{\left(k\right)}-Ax^{\left(k+1\right)}\right\|}_{2}^{2}\leq 2\lambda (L_{q}(x^{\left(k\right)},\varepsilon_{k},\lambda )-L_{q}(x^{\left(k+1\right)},\varepsilon_{k+1},\lambda ))$$

Furthermore,

(18) $${\left\|x^{\left(k+1\right)}-x^{\left(k\right)}\right\|}_{2}^{2}\leq C_{4}(L_{q}(x^{\left(k\right)},\varepsilon_{k},\lambda )-L_{q}(x^{\left(k+1\right)},\varepsilon_{k+1},\lambda ))$$

where *C*_4_ is an independent positive constant.

**Proof.**  We first compute the following formula,

(19) $$\begin{array}{l} L_{q}(x^{\left(k\right)},\varepsilon_{k},\lambda )-L_{q}(x^{\left(k+1\right)},\varepsilon_{k+1},\lambda )\\ =\sum_{j=1}^{N}{\left(\varepsilon_{k}^{2}+{\left|x_{j}^{\left(k\right)}\right|}^{2}\right)}^{\frac{q}{2}}-\sum_{j=1}^{N}{\left(\varepsilon_{k+1}^{2}+{\left|x_{j}^{\left(k+1\right)}\right|}^{2}\right)}^{\frac{q}{2}}+\frac{1}{2\lambda }({\left\|Ax^{\left(k\right)}-b\right\|}_{2}^{2}-{\left\|Ax^{\left(k+1\right)}-b\right\|}_{2}^{2})\\ =\sum_{j=1}^{N}{\left(\varepsilon_{k}^{2}+{\left|x_{j}^{\left(k\right)}\right|}^{2}\right)}^{\frac{q}{2}}-{\left(\varepsilon_{k+1}^{2}+{\left|x_{j}^{\left(k+1\right)}\right|}^{2}\right)}^{\frac{q}{2}}+\frac{1}{2\lambda }{\left\|Ax^{\left(k\right)}-Ax^{\left(k+1\right)}\right\|}_{2}^{2}\\ +\frac{1}{\lambda }{\left(Ax^{\left(k+1\right)}-b\right)}^{T}(Ax^{\left(k\right)}-Ax^{\left(k+1\right)}) \end{array}$$

According to ${\left[\frac{q\alpha^{\left(k+1\right)}[i]}{{\left(\varepsilon_{k}^{2}+{\left\|\alpha^{\left(k\right)}[i]\right\|}_{2}^{2}\right)}^{1-\frac{q}{2}}}\right]}_{1\leq i\leq M}+\frac{1}{\lambda }A^{T}(A\alpha^{\left(k+1\right)}-b)=0$, we have

(20) $$\frac{1}{\lambda }{\left(Ax^{\left(k+1\right)}-b\right)}^{T}(Ax^{\left(k\right)}-Ax^{\left(k+1\right)})=-\sum_{j=1}^{N}\frac{qx_{j}^{\left(k+1\right)}(x_{j}^{\left(k\right)}-x_{j}^{\left(k+!\right)})}{{\left(\varepsilon_{k}^{2}+{\left|x_{j}^{\left(k\right)}\right|}^{2}\right)}^{1-\frac{q}{2}}}$$

Using Lemma 1 and substituting Equation (20) to Equation (19), we have

(21) $$\begin{array}{l} L_{q}(x^{\left(k\right)},\varepsilon_{k},\lambda )-L_{q}(x^{\left(k+1\right)},\varepsilon_{k+1},\lambda )\\ =\sum_{j=1}^{N}\{{\left(\varepsilon_{k}^{2}+{\left|x_{j}^{\left(k\right)}\right|}^{2}\right)}^{\frac{q}{2}}-{\left(\varepsilon_{k+1}^{2}+{\left|x_{j}^{\left(k+1\right)}\right|}^{2}\right)}^{\frac{q}{2}}-\frac{qx_{j}^{\left(k+1\right)}(x_{j}^{\left(k\right)}-x_{j}^{\left(k+!\right)})}{{\left(\varepsilon_{k}^{2}+{\left|x_{j}^{\left(k\right)}\right|}^{2}\right)}^{1-\frac{q}{2}}}\}\\ +\frac{1}{2\lambda }{\left\|Ax^{\left(k\right)}-Ax^{\left(k+1\right)}\right\|}_{2}^{2}\\ \geq \frac{1}{2\lambda }{\left\|Ax^{\left(k\right)}-Ax^{\left(k+1\right)}\right\|}_{2}^{2}+\sum_{j=1}^{N}{\left(x_{j}^{\left(k\right)}-x_{j}^{\left(k+1\right)}\right)}^{2}\frac{q}{2{\left(\varepsilon_{k}^{2}+{\left|x_{j}^{\left(k\right)}\right|}^{2}\right)}^{1-\frac{q}{2}}} \end{array}$$

From the result of Equation (21), the Equation (17) can be calculated.

It should be noted from Equation (17) that the $L_{q}(x^{\left(k\right)},\varepsilon_{k},\lambda )$ is monotonically decreasing sequence, hence,

(22) $${\left\|x^{\left(k\right)}\right\|}_{q}^{q}\leq {\left\|x^{\left(k\right)}\right\|}_{q,\varepsilon_{k}}^{q}\leq L_{q}(x^{\left(k\right)},\varepsilon_{k},\lambda )\leq L_{q}(x^{\left(0\right)},\varepsilon_{0},\lambda )={\left\|x^{\left(0\right)}\right\|}_{q,\varepsilon_{0}}^{q}$$

for all *k* ≥ 1 and 1 ≤ *i* ≤ *n*, there exists a positive number β which satisfies ${\left\|x^{\left(k\right)}\right\|}_{\infty }\leq \beta$, hence

(23) $$\frac{q}{2{\left(\varepsilon_{k}+{\left|x_{j}^{\left(k\right)}\right|}^{2}\right)}^{1-\frac{q}{2}}}\geq \frac{q}{2{\left(\varepsilon_{0}+\beta^{2}\right)}^{1-\frac{q}{2}}}$$

Let $\frac{1}{C_{4}}=\frac{q}{2{\left(\varepsilon_{0}+\beta^{2}\right)}^{1-\frac{q}{2}}}$, and the Lemma 2 is proved conclusively. ☐

Herein, combining the above inequalities in Lemma 1 and Lemma 2, the Theorem 1 can be proved ultimately, for simplicity, the detailed proof process was omitted. In the next section, the choice of regular operator *q* will be discussed in detail via a simulation experiment.

**Figure 1:**
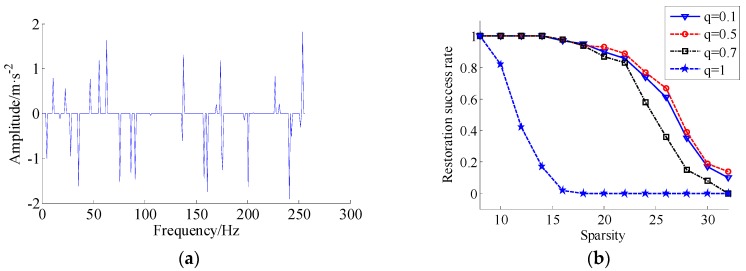
Random impulses and the comparison results of recoverability with different *q*. (**a**) Random signal with 32 non-zero impulses; (**b**) The comparison results of recoverability with different *q*.

For the choice of regular operator *q* (0 < *q* ≤ 1), let *q* varying among {0.1, 0.5, 0.7, 1}. Firstly, the matrix *A* is designed by rand-function rand(64, 256) in MATLAB, and the initialization signal $x^{0}$ has *t* non-zero narrow impulses that subject to the standard Gaussian distribution (SGD), the locations of non-zeros are uniformly and randomly generated, and *t*, varying among {8, 10, 12, …, 32}. The penalty parameter $\lambda =10^{-6}$ is small enough to approximately enforce $Ax=Ax^{0}$ and δ=0.09, which is measured over 100 times in terms the perfect reconstruction. Taking the SPSR algorithm iterative 1000 times, if the recovery error satisfies ${\left\|x^{r}-x^{0}\right\|}_{2}/{\left\|x^{0}\right\|}_{2}\leq 10^{-3}$, the iteration is stopped, where $x^{r}$ stands for a recovered vector. The recovery algorithm is SPSR method (*q* ∈ {0.1, 0.5, 0.7, 1}), the recovery success rate curve of different. *q* with sparsity is shown in Figure 1. From Figure 1, it can be seen that *q* = 0.1, *q* = 0.5 performed better than *q* = 0.7 and much better than *q* = 1. Moreover, *q* = 0.5 gives a higher success than *q* = 0.1 slightly. We emphasize that our results do not counter the intuition that a smaller *q* should recover more sparse vectors. Generally, a smaller *q* value makes the minimizing functional more non-convex, but more difficult to solve. In addition, in this algorithm, it has been discovered that if smoothing parameter ε decreased slowly, the performance of *q* = 0.1 reach further improved. However, the running time with *q* = 0.1 also became much longer. For example, in this simulation, the execution time of parameter *q* = 0.5 is 4.4550 s, and execution time of *q* = 0.7 and *q* = 1 are 5.7359 s and 8.6348 s, respectively, but the execution time of *q* = 0.1 is 9.4643 s, it should be noted that the execution time becoming longer when parameter q is selected smaller.

## 4. Case Verifications and Disscussion

In order to quantitatively calculate restoration effect, three restoration performance standards (RPSs) are employed for comparison, i.e., the normalized mean-square error (NMSE), peak signal-to-noise ratio (PSNR), and structural similarity (SSIM), respectively. It should be noted that the PSNR reflects the approximation degree between the recovered image and original image, and the SSIM reflects the similarity degree between the recovered image and original image. Three RPSs are defined as

(24) $$NMSE=\frac{\sum {\left[x-\overset{\wedge }{x}\right]}^{2}}{\sum {\left[x-\bar{x}\right]}^{2}}$$

(25) $$PSNR=20\times \mathrm{lg}\frac{255^{2}}{{\left\|x-\overset{\wedge }{x}\right\|}_{2}^{2}}$$

where *x* is an original image and $\overset{\wedge }{x}$ is a recovered image.

(26) $$SSIM=\frac{\left(2\mu_{x}\mu_{\overset{\wedge }{x}}+c_{1})(2\sigma_{x,\overset{\wedge }{x}}+c_{2}\right)}{\left(\mu_{x}^{2}+\mu_{\overset{\wedge }{x}}^{2}+c_{1})(\sigma_{x}^{2}+\sigma_{\overset{\wedge }{x}}^{2}+c_{2}\right)}$$

where μ is the mean of image, σ is variance or covariance, *c*_1_ and *c*_2_ is the small constant which can enhance the stability of calculation results, respectively.

In the following experiments, the microstructure image is divided into 8 × 8 blocks. The DCT dictionary is used as initial dictionary, and KSVD is used as dictionary training algorithm, then the damaged microstructure images are applied as training samples. The KSVD dictionary training parameters setting is shown in Table 1.

### 4.1. Simulation Case of Aluminum Alloy 7075 Material

**Figure 2:**
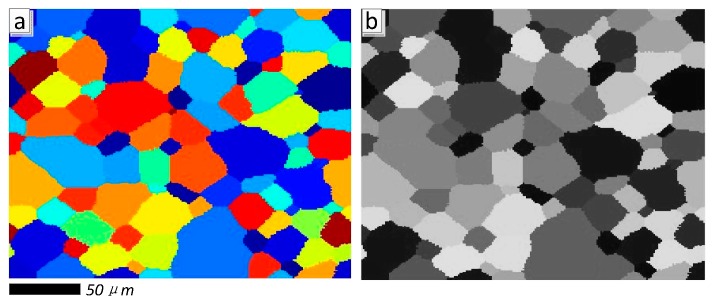
The ideal microstructure images of aluminum alloy 7075 material generated by cellular automaton simulation (CAS) method. (**a**) Ideal color image; (**b**) the grayscale image.

**Figure 3:**
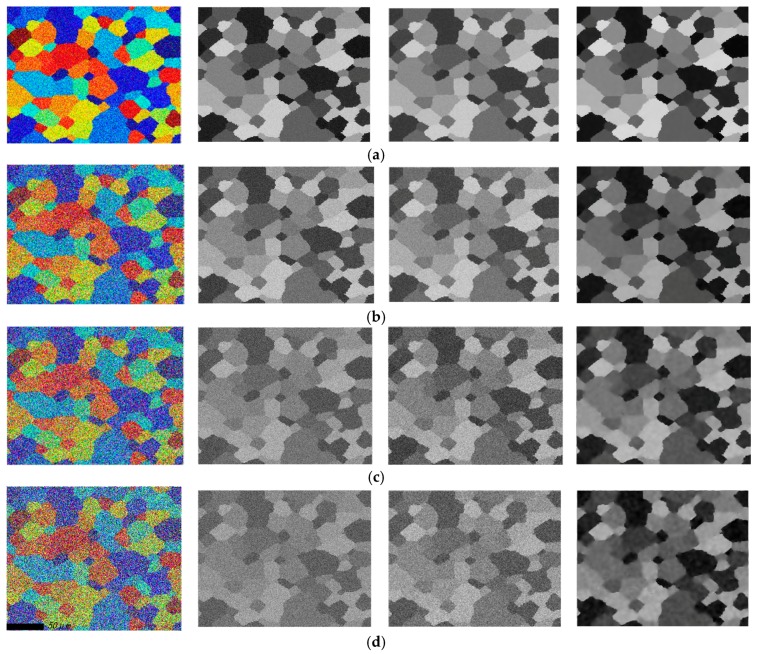
Example orientation image with Gaussian noise added and orientation images obtained after conventional wavelet packet and the proposed SPSR (*q* = 0.5) with noise levels of (**a**) 0.1; (**b**) 0.5; (**c**) 0.8; and (**d**) 1.0. (from top row to bottom row); Noised orientation images of the color, grayscale, images recovered by wavelet packet method, images recovered by SPSR (*q* = 0.5) (from left column to right column).

To verify the superiority of proposed SPSR (*q* = 0.5) approach, an ideal microstructure orientation image of aluminum alloy 7075 material is generated by cellular automaton simulation (CAS) method [42,43], see Figure 2, Figure 2a is the ideal color image, and Figure 2b is the grayscale image, respectively. The artificial Gaussian noises are added in the ideal microstructure image, and Figure 3 presents the visual quality of the reconstructed images based on wavelet packet and SPSR (here, *q* = 0.5) algorithms when the noise level is fixed on 0.1, 0.5, 0.8, and 1.0, respectively. It can be seen from Figure 3 that the proposed SPSR (*q* = 0.5) method clearly provides a significant improvement to the fidelity of the resultant image (see the 4th column). The wavelet packet method is also applied to process the noisy orientation image (see the 3rd column), and provides some improvement, but not nearly as dramatic as that achieved with the SPSR method. More importantly, as we can see, the proposed SPSR method achieves a significantly better visual quality than wavelet packet method in smooth regions.

Table 2 shows quantitative results of the restoration performance standards (RPSs) obtained for the images shown in Figure 3. It can be see that the wavelet-packet method does provide a moderate improvement particularly at the higher noise levels as evidenced by Figure 3. Further, as the images show as well, the SPSR (*q* = 0.5) method provides much more significant improvement in RPSs. It should be noted that adding more noise to the orientation image lead to much higher NMSE, lower PSNR and SSIM, and the data did not follow the expected trend, likely due to so many missing points.

In the next two sections, two kinds of damaged aluminum alloy 7075 (AA7075) orientation images are investigated. The first damaged image is contaminated, due to the charged particles and dust that exists in the electron back-scattered diffraction (EBSD) system. The second damaged image is a low-pixel (or low-resolution) image that is contaminated due to the thicker contamination membrane residues on the AA7075 sample surface. The orientation images were acquired by the Oxford Instruments AZtecHKL EBSD system. Orientation images were collected and recorded at 114 × 114 pixel. The Hough was run on the patterns after compression to 96 × 96 pixels with a 9 × 9 convolution mask, 1° theta step size, and searching for a maximum of 10 peaks.

### 4.2. Experimental Case 1

**Figure 4:**
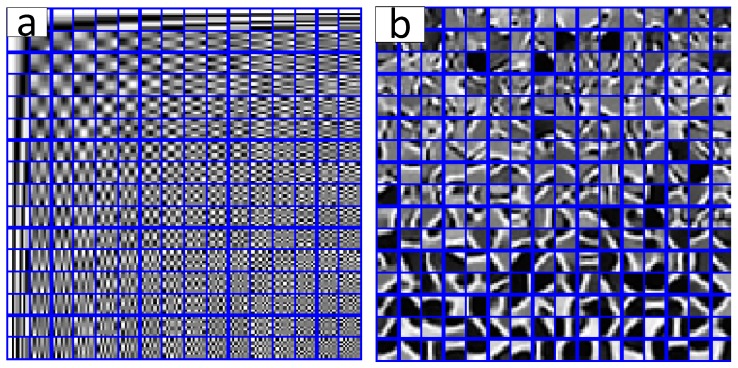
The discrete cosine transform (DCT) dictionary and KSVD dictionary of first damaged image. (**a**) The DCT dictionary; (**b**) The KSVD dictionary (iterated for 25 times).

The initial DCT dictionary of the first damaged image and the over-complete dictionary that was trained by KSVD algorithm are shown in Figure 4a,b, respectively. It is obvious that the structure of KSVD dictionary is more abundant than the DCT dictionary, the reason is that the KSVD dictionary fully reflects the detailed structure characteristics of the image, equivalently, the KSVD dictionary can precisely represent the sparse characterization of block sub-image.

**Figure 5:**
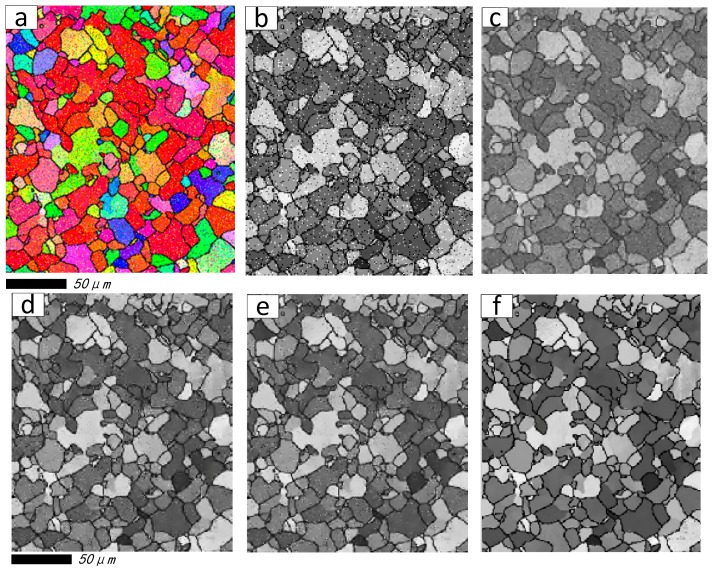
Restoration results of the first damaged image. (**a**) Color damaged image; (**b**) Grayscale damaged image; (**c**) Restoration result generated by wavelet packet method; (**d**) Restoration result generated by DCT + orthogonal matching pursuit (OMP) method; (**e**) Restoration result generated by KSVD + OMP method; (**f**) Restoration result generated by proposed method.

Figure 5a,b are the color damaged image and its grayscale image. The reconstructed results generated by wavelet packet method, the DCT combined with OMP method (namely DCT + OMP), the KSVD combined with OMP method (namely KSVD + OMP), and the proposed method (namely KSVD + SPSR, *q* = 0.5) are illustrated in Figure 5c, Figure 5d, Figure 5e, and Figure 5f, respectively. From Figure 5c–f, it can be observed that all the reconstruction methods are able to reasonably recover the damaged image.

The quantitative RPSs between the actual and recovered image based on the above methods are summarized in Table 3. In this work, due to the ideal image being unknown, the above RPS results are designed based on damaged and recovered image, therefore, it should be noted that the larger the value of NMSE, the smaller the values of PSNR and SSIM, and the more accurate of restoration effect.

Form Table 3, it is clear that the proposed approach achieves better restoration result than other methods. In the smooth regions, through the proposed approach, one can find that the clarity and fidelity of image grain-boundary are enhanced significantly, and the influence of the speckle noise is reduced greatly.

**Figure 6:**
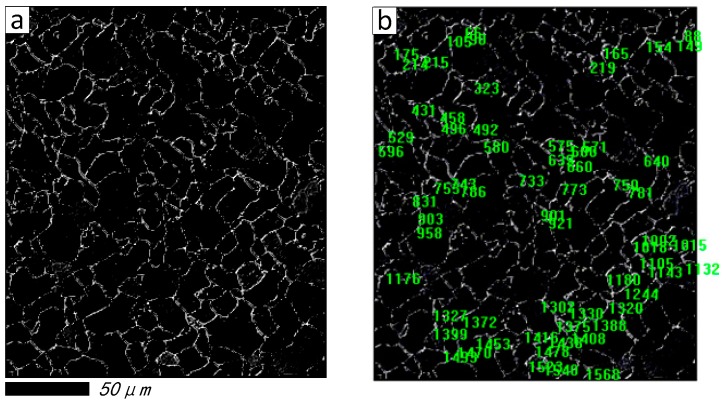
Segmentation effect and its statistical result before restored. (**a**) Segmentation effect; (**b**) Grain number tags.

**Figure 7:**
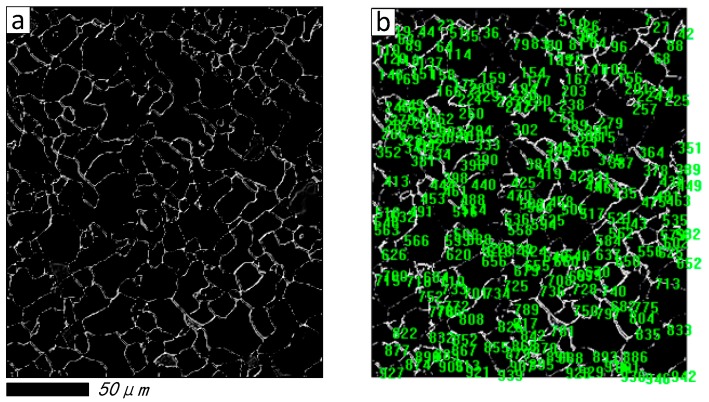
Segmentation effect and its statistical result restored by proposed method. (**a**) Segmentation effect; (**b**) Grain number tags.

Furthermore, to explore the grain distribution and its statistical result before and after restoration, the maximum entropy threshold segmentation (METS) algorithm [44] is introduced in Figure 5b,f, respectively. The segmentation effect and grain number tags created by image pro-plus software [45] are shown in Figure 6 and Figure 7, respectively. It is obvious that breakpoints of segmentation curves in Figure 7a are less than Figure 6a, and the number of grains in Figure 7b is more than Figure 6b.

Table 4 summarizes the statistical results of grain size, which consists of grain diameter (mean), grain area (polygon) and grain perimeter (ratio). It can be seen that the sample number is 64 before being restored, and it has increased rapidly to 232 after being restored by the proposed method; this is because more grains are calculated, due to the sealing character of the segmentation curves in Figure 7b.

**Figure 8:**
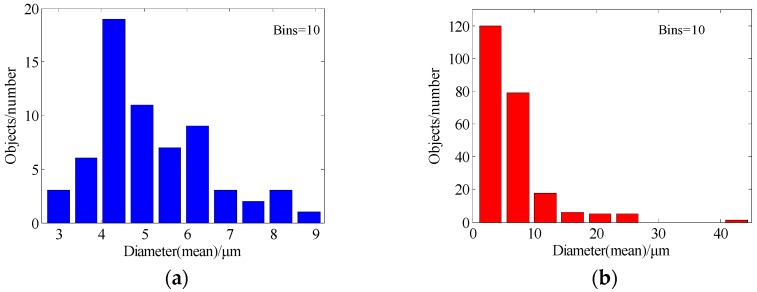
The grain diameter (mean) histogram. (**a**) Before restoration; (**b**) After restoration by proposed method.

**Figure 9:**
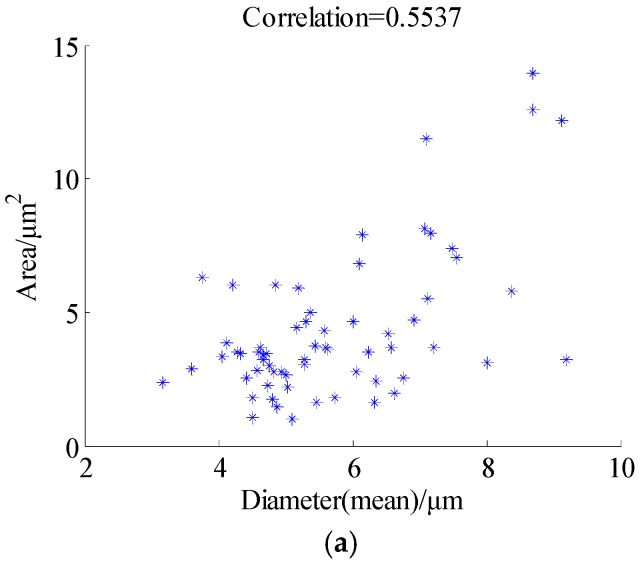

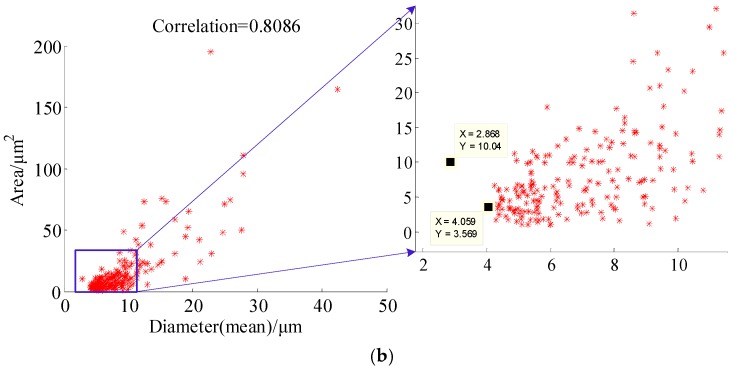
The scatter diagram of grain diameter (mean) and grain area. (**a**) Before restoration; (**b**) After restoration by proposed method.

Figure 8 illustrates the histogram distribution of grain diameter, and Figure 9 shows the relationship between the grain diameter and grain area before and after being restored, respectively. From Figure 8a, it should be noted that the grain number is not counted when the grain size larger than 10 microns, because most of the grain boundaries are broken in the original damaged image, and only some continuous grains will be counted by the image pro-plus software; on the contrary, the grain can be calculated easily based upon the proposed method. From the results shown in Figure 9, the correlation value is 0.5537 in Figure 9a, and Figure 9b is 0.8086. From the scatter diagram of grain diameter (mean) after being restored by proposed method, it can be seen that the minimum value of the grain diameter (mean) is 2.868 μm, and grain area is 10.04 μm^2^, and most of the grains with diameters below 10 μm can be detected. It is proven that the grain distribution in Figure 6b is more dispersed than Figure 7b. Overall, it concluded from the experiments that, by both visual comparison and statistical assessment, the proposed method shows better restoration performance compared with some state-of-the-art methods.

### 4.3. Experimental Case 2

**Figure 10:**
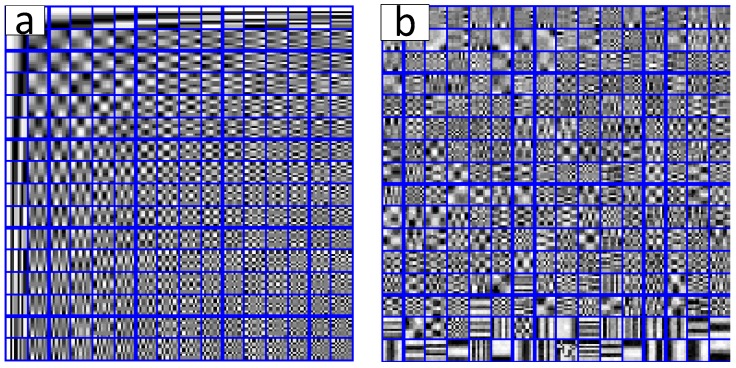
The DCT dictionary and KSVD dictionary of second damaged image. (**a**) The DCT dictionary; (**b**) The KSVD dictionary (iterated 25 times).

For the low pixel (or low resolution) image, usually, in the engineering application, it is difficult to test the metal macro-mechanical property based on the existing grain boundary and dislocation orientation. Similar to the above experimental steps, the KSVD algorithm is applied to the DCT initial dictionary, and the training parameter setting coincided with Table 1. The DCT dictionary and trained KSVD dictionary are presented in Figure 10a and Figure 10b, respectively.

**Figure 11:**
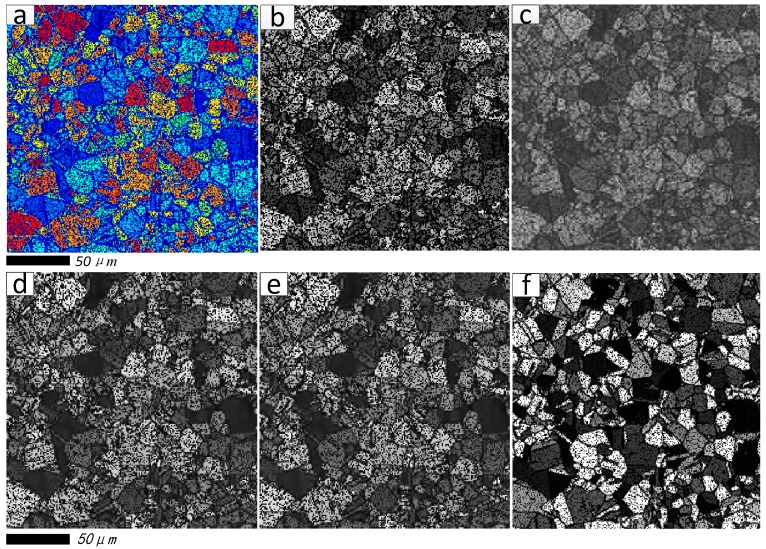
Restoration results of the second damaged image. (**a**) Color-damaged image; (**b**) Grayscale damaged image; (**c**) Restoration result generated by wavelet packet method; (**d**) Restoration result generated by DCT + OMP method; (**e**) Restoration result generated by KSVD + OMP method; (**f**) Restoration result generated by the proposed method.

Figure 11a,b are color-damaged image and its grayscale image. The comparison results of the wavelet packet, the DCT + OMP, the KSVD + OMP, and the proposed methods, are illustrated in Figure 11c, Figure 11d, Figure 11e, and Figure 11f, respectively. Three RPSs are displayed in the Table 5. Unfortunately, from Figure 11c and Table 5, it is obvious that the grain boundaries in recovered images are more unclear than the damaged image, based on the wavelet packet method. As shown in the close-up views of Figure 11d,e, KSVD + OMP leads to better restoration of edges and fewer artifacts than DCT + OMP, that is, because the KSVD dictionary contains more detailed structural information than the DCT dictionary. More importantly, the proposed algorithm still outperforms the other three methods; as demonstrated in Figure 11f, the number of black artifacts is reduced dramatically by the proposed method, and the grain boundaries of multi-regions become more legible.

**Figure 12:**
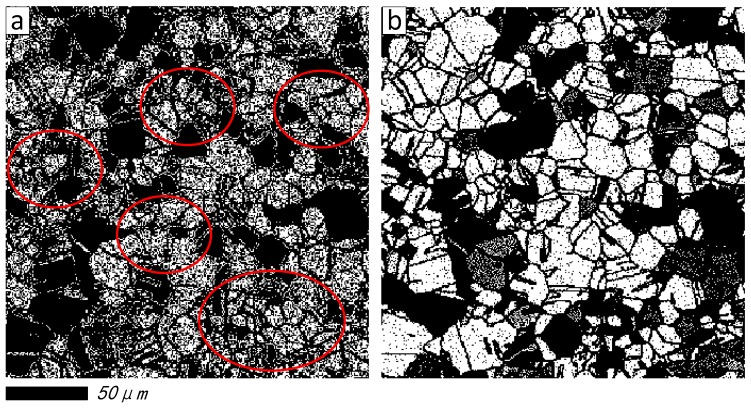
The comparison of grain boundary of the second damaged image. (**a**) Before restoration; (**b**) After restoration by proposed method.

Furthermore, in order to discuss the restoration effect on grain boundary, the image segmentation and edge detection methods are applied on Figure 11b,f. Figure 12 shows the comparison of grain boundaries for the second damaged image. As illustrated in Figure 12a, the grain boundaries with small size (see the red circle) are still relatively obscure, and the grain number cannot be estimated accurately. After recovery via the proposed algorithm, the grain boundaries and grain number have a greater improvement, and are increasing. Compared with Figure 12a, it should be noted from Figure 12b that a larger number of continuous lamellar (plate-like) structures are recovered, and some breakpoints are reconnected by the proposed method, image segmentation, and edge detection method, meanwhile, the grain number will be increased via the image pro-plus software. Due to that the ideal microstructure image is unknown, comparing Figure 11 and Figure 12, some data that may not show in ideal microstructure image might be created (thus over-correcting), this phenomenon might be improved by adjusting parameter q. Generally, for the SPSR method, the reconstruction effect and sharpness degree will be further improved when the regular operator q that is selected is much smaller, such as *q* = 0.1. However, the running time with *q* = 0.1 also became much longer. In this work, the regular operator q is selected to 0.5, as one can find in Table 5, that the running time of the proposed method (3371.2670 s) is much longer than wavelet packet (0.4969) and other sparse representation methods (DCT + OMP is 165.4376 s, and KSVD + OMP is 2928.2968 s) due to dictionary training and its iteration operations, thus, the faster calculation method based on adaptive selection method of regular operator q will be explored in future studies for eliminating the drawback of over-correcting.

## 5. Conclusions

To address the image restoration problem, a new image reconstruction technique for microstructure image based on KSVD and smoothing penalty sparse representation (SPSR) algorithm is proposed in this paper. In image sparse representation, traditional orthogonal basis functions are replaced by trained KSVD dictionary, and the trained KSVD dictionary represents the sparse characterization of block sub-image probably due to the traditional sparse representation methods that may cause instability and obvious artifacts in the reconstructed image, especially for the restoration of microstructure images, including some smooth regions, or when the noise level is strong. The proposed algorithm can overcome the above issues, which improves the reconstruction accuracy significantly. Moreover, the damaged microstructure image usually brings statistics missing in the analysis of microstructure grain parameters, and the proposed method can effectively address this drawback, which has a high engineering application value in metal materials, manufacturing, and microstructure fields.

Although the proposed method improves the reconstruction quality significantly, it still needs future improvements, where the complexity level and computational time of the proposed approach is rather high, due to dictionary training and its iteration operations. It is suggested that faster calculation methods will be explored in future studies.

## Figures and Tables

**Table 1:** Parameters setting of K-means singular value decomposition (KSVD) algorithm.

Training Sample Size	Redundancy	Dictionary Size	Iterations
8 × 8	6	64 × 256	25

**Table 2:** The restoration performance standard (RPS) results of wavelet-packet and proposed method.

Inpainting Methods	Noise Level	NMSE	PSNR/dB	SSIM
**Wavelet-packet**	0.1	1.8650	24.9068	0.9784
	0.5	2.9994	22.8410	0.9648
	0.8	6.9625	19.1669	0.9197
	1	13.6314	16.2595	0.8530
**SPSR (*q* = 0.5)**	0.1	0.1241	36.5150	0.9984
	0.5	0.3616	31.8698	0.9954
	0.8	1.0988	27.0495	0.9859
	1	1.9265	24.5989	0.9748

**Table 3:** The RPSs comparison of the first damaged image.

Image	Restoration Methods	NMSE	PSNR/dB	SSIM	Time (s)
-	Wavelet packet	4.0825	21.4212	0.9345	0.6172
**First damaged image**	DCT + OMP	2.3295	23.6980	0.9587	52.9017
-	KSVD + OMP	2.3458	23.6679	0.9585	705.3599
-	Proposed method	6.8192	19.0388	0.8953	1329.7895

**Table 4:** Statistical parameters of grain size of the first damaged image.

Statistic	Diameter (Mean)/μm		Area (Polygon)/μm^2^		Perimeter (Ratio)/μm	
	Before	After	Before	After	Before	After
**Min**	3.1622	2.8680	1	1	0.4516	0.5589
**Max**	9.1923	42.3221	13.9444	194.7222	1	1
**Mean**	5.7244	8.6099	4.3257	14.8863	0.8873	0.8635
**Grain number**	64	232	64	232	64	232

**Table 5:** The RPSs comparison of the second damaged image.

Image	Restoration Methods	NMSE	PSNR/dB	SSIM	Time (s)
-	Wavelet packet	33.6264	14.0288	0.7017	0.4969
**Second damaged image**	DCT + OMP	73.8015	11.1316	0.5523	165.4376
-	KSVD + OMP	74.1460	11.1111	0.5499	2928.2968
-	Proposed method	85.2766	10.3924	0.5131	3371.2670

## Nomenclature

*y*	Damaged image
*x*	Latent ideal image
*n*	External noise
*R*	Degradation matrix
φ	Transformation domain
α	Transformation coefficients
$x_{0}$	1-D discrete signal
*A*	Sensing matrix
*D*	Over-complete dictionary
*d_k_*	The *k*-th column of the pending update dictionary
***E**_k_*	Pre-computed error matrix
$\Omega_{k}$	Matrix with size $N\times \left|\omega_{k}\right|$
$E_{k}^{R}$	Error columns
λ	Regularization weight
ζ(x)	Regularization term
*q*	Regular operator
ε	Smoothing parameter
*s*	Estimated sparsity level
*b*	Measurement vector
*β*	Constant
*C*_1_, *C*_2_, *C*_3_	Independent positive constants
KSVD	K-means singular value decomposition
SPSR	Smoothing penalty sparse representation
AA7075	Aluminum alloy 7075
STM	Surface topography measurement
MEMM	Micro-electro mechanical manufacturing
AFD	Adaptive filter denoising
W-WPD	Wavelet–wavelet packet denoising
PDE	Partial differential equation
WHMRF	Wavelet hidden Markov random field
SR	Sparse representation
SAR	Synthetic aperture radar
TIBT	Translation-invariant second-generation band-wavelet transform
DCT	Discrete cosine transform
NP hard	Non-deterministic polynomial-time hard
MP	Matching pursuit
OMP	Orthogonal matching pursuit
CoSaMP	Compressive sampling matching pursuit
TVRF	Total variation regularization function
SNLR	Sparse non-local regularization
CS	Compressed sensing
DWT	Discrete Wavelet transform
RIP	Restricted isometry property
GRM	Gaussian Random matrix
LFM	Local Fourier matrix
LHM	Local Hadamard matrix
HUE	Highly underdetermined equation
SGD	Standard Gaussian distribution
RPSs	Restoration performance standards
NMSE	Normalized mean-square error
PSNR	Peak signal-to-noise ratio
SSIM	Structural similarity
CAS	Cellular automaton simulation
EBSD	Electron back-scattered diffraction
METS	Maximum entropy threshold segmentation
