# Correction: Li, Q. and Liang, S.Y. Microstructure Images Restoration of Metallic Materials Based upon KSVD and Smoothing Penalty Sparse Representation Approach. *Materials* 2018, *11*, 637

**DOI:** 10.3390/ma13112436

**Published:** 2020-05-26

**Authors:** Qing Li, Steven Y. Liang

**Affiliations:** 1 College of Mechanical Engineering, Donghua University, Shanghai 201620, China; steven.liang@me.gatech.edu; 2 George W. Woodruff School of Mechanical Engineering, Georgia Institute of Technology, Atlanta, GA 30332-0405, USA

The authors were not aware of some errors and imprecise descriptions made in the proofreading phase, therefore, we wish to make the following corrections to this paper [1]. These changes do not affect the scientific results and conclusions. 

In page 1, Section 1, paragraph 1, the word ”re-cquisition” should be read as “re-acquisition”. 

In page 4, Section 2.2, paragraph 1, the sentence “The adaptive over-complete KSVD dictionary method is designed as follows” should be read as “The adaptive over-complete KSVD dictionary method is designed as follows [16,36]”. 

In page 5, Section 3, the corrected second paragraph reads “To overcome the above issue, inspired by the ideas of the unconstrained low-rank matrix recovery in refs. [40–42] that have been implemented in the compressed sensing field [31–33], a novel smoothing penalty sparse representation (SPSR) method is introduced, which is different from the ones studied in refs. [40–42] where a uniform random matrix (i.e., the entries of the matrix are random variables with uniform distribution) was used. In this work, the matrix is obtained via the mutual coherence technique [37] and over-complete KSVD dictionary that satisfies the RIP criterion. The objective function is as follows,”.

40. Lai, M.J.; Wang, J.Y. An unconstrained *l_q_* minimization with 0 < *q* ≤ 1 for sparse solution of underdetermined linear systems. *SIAM J. Optim*. **2011**, *21*, 82–101.
41. Lai, M.J.; Xu, Y.Y.; Yin, W.T. Improved iteratively reweighted least squares for unconstrained smoothed lq minimization. *SIAM J. Numer. Anal*. **2013**, *51*, 927–957.
42. Wang, Y.; Wang, J.; Xu, Z. On recovery of block-sparse signals via mixed *l_2_*/*l_q_* (0 < *q* ≤ 1) norm minimization. *EURASIP J. Adv. Signal Process.* **2013**, *76*.

In page 6, the corrected Equation (16) is 

“${\left(\varepsilon_{k}^{2}+{\left\|x\right\|}_{2}^{2}\right)}^{1-q/2}{\left(\varepsilon_{k+1}^{2}+{\left\|y\right\|}_{2}^{2}\right)}^{q/2}\leq (1-\frac{q}{2})(\varepsilon_{k}^{2}+{\left\|x\right\|}_{2}^{2})+\frac{q}{2}(\varepsilon_{k+1}^{2}+{\left\|y\right\|}_{2}^{2})$”. 

In page 6, the sentence “To prove the theorem 1, the following two lemmas are required” should be read as: “To prove the theorem 1, the following two lemmas (i.e., lemma 1 and lemma 2) [40,41] are required”. The titles “Lemma 1 and its proof” and “Lemma 2 and its proof” should be read as “Lemma 1 [40,41]”, “proof [40,41]” and “Lemma 2 [40,41]”, “proof [40,41]”. 

In pages 8, the corrected Equation (23) is ”$\frac{q}{2{\left(\varepsilon_{k}^{2}+{\left|x_{j}^{\left(k\right)}\right|}^{2}\right)}^{1-q/2}}\geq \frac{q}{2{\left(\varepsilon_{0}^{2}+\beta^{2}\right)}^{1-q/2}}$”, and after Equation (23), $\frac{1}{C_{4}}=\frac{q}{2{\left(\varepsilon_{0}^{2}+\beta^{2}\right)}^{1-q/2}}$. 

In page 11, Section 4.1, final paragraph, the sentence “The first damaged image is contaminated, due to the charged particles and dust that exists in the electron back-scattered diffraction (EBSD) system. The second damaged image is a low-pixel (or low-resolution) image that is contaminated due to the thicker contamination membrane residues on the AA7075 sample surface. The orientation images were acquired by the Oxford Instruments AZtecHKL EBSD system” should be read as “The first damaged image is contaminated, due to the charged particles and dust that exists in the electron back-scattered diffraction (EBSD) system, which is artificially contaminated via adding Gaussian and impulse noises. The second damaged image is a low-pixel (or low-resolution) image that is contaminated due to the thicker contamination membrane residues on the sample surface, which is artificially contaminated via pixel masking operation. The orientation images were acquired by the Oxford Instruments AZtecHKL EBSD system (see open website www.ebsd.cn)”. 

Due to the addition of new references [40–42], the order of the original citations [40–45] should be changed with [43–48], correspondingly. 

## References

[1] Li Q., Liang S.Y. (2018). Microstructure Images Restoration of Metallic Materials Based upon KSVD and Smoothing Penalty Sparse Representation Approach. Materials.

